# Clinical Profiles and CMR Findings of Young Adults and Pediatrics with Acute Myocarditis Following mRNA COVID-19 Vaccination: A Case Series

**DOI:** 10.3390/vaccines10020169

**Published:** 2022-01-22

**Authors:** Roberto Manfredi, Francesco Bianco, Valentina Bucciarelli, Giuseppe Ciliberti, Federico Guerra, Nicolò Schicchi, Marcello Tavio, Emanuela Berton, Francesca Chiara Surace, Massimo Colaneri, Sabina Gallina, Marco Pozzi

**Affiliations:** 1 Cardiology and Arrhythmology Clinic, University Hospital “Umberto I-Lancisi-Salesi”, Marche Polytechnic University, 60123 Ancona, Italy; r.manfredi@pm.univpm.it (R.M.); giuseppe.ciliberti@ospedaliriuniti.marche.it (G.C.); f.guerra@univpm.it (F.G.); 2 Department of Pediatric and Congenital Cardiology and Cardiac Surgery—Azienda Ospedaliero, Universitaria “Ospedali Riuniti” di Ancona, 60123 Ancona, Italy; valentina.bucciarelli@ospedaliriuniti.marche.it (V.B.); emanuela.berton@ospedaliriuniti.marche.it (E.B.); francescachiara.surace@ospedaliriuniti.marche.it (F.C.S.); massimo.colaneri@ospedaliriuniti.marche.it (M.C.); marco.pozzi@ospedaliriuniti.marche.it (M.P.); 3 Radiology Department, Azienda Ospedaliero Universitaria “Ospedali Riuniti”, 60123 Ancona, Italy; nicolo.schicchi@ospedaliriuniti.marche.it; 4 Unit of Emerging and Immunosuppressed Infectious Diseases, Department of Gastroenterology and Transplantation, Polytechnic University of Marche, 60123 Ancona, Italy; marcello.tavio@ospedaliriuniti.marche.it; 5 Department of Neurosciences, Imaging and Clinical Sciences, Gabriele d’Annunzio University of Chieti-Pescara, 66100 Chieti, Italy; sgallina@unich.it

**Keywords:** COVID-19, myocarditis, myopericarditis, COVID-19 vaccination, COVID-19 vaccines

## Abstract

Messenger RNA (mRNA) coronavirus disease of 2019 (COVID-19) vaccines have been recently associated with acute myocarditis, predominantly in healthy young males. Out of 231,989 vaccines administrated in our region (Marche, Italy), we report a case series of six healthy patients (four males and two females, 16.5 years old (Q1, Q3: 15, 18)) that experienced mRNA-COVID-19-vaccines side effects. All patients were hospitalized due to fever and troponins elevation following the second dose of an mRNA-based COVID-19 vaccine. Cardiovascular magnetic resonance (CMR) was performed 72–96 h after vaccination. All patients were treated with colchicine and ibuprofen. Myocarditis was prevalent in males. It was characterized by myocardial edema and late gadolinium enhancement (LGE) in the lateral wall of the left ventricle (LV). One patient showed sole right ventricular involvement, while the females presented with myopericarditis (myocarditis + pericardial effusion). All patients in our series had preserved LV ejection fraction and remained clinically stable during a relatively short inpatient hospital stay. One case presented with atrial tachycardia. At the follow-up, no significant CMR findings were documented after a three-month medical treatment. According to other recently published case series, our report suggests a possible association between acute myocarditis and myopericarditis with mRNA COVID-19 vaccination in healthy young adults and pediatric patients. Not only males are involved, while some arrhythmic manifestations are possible, such as atrial tachycardia. Conversely, we here highlight the benign nature of such complications and the absence of CMR findings after a three-month medical treatment with colchicine and ibuprofen.

## 1. Introduction

Since the coronavirus disease of 2019 (COVID-19) outbreak, severe acute respiratory syndrome coronavirus 2 (SARS-CoV-2) involved about 169 million people worldwide, resulting in more than 3.5 million deaths [1]. In December 2020, the food and drug administration (FDA) and the European Medicines Agency (EMA) approved two messenger RNA (mRNA) vaccines (Pfizer-BioNTech and Moderna) to reduce the risk and severity of COVID-19 infection [2]. Firstly, they were administrated exclusively in the adults. Then, the FDA and EMA extended the mRNA COVID-19 vaccines utilization to ≥12 years old patients, with nearly 1,548,000 doses administrated in the pediatric population only in the United States (US) [3,4].

On 17 May 2021, the US Centers for Disease Control and Prevention (CDC) conveyed various cases of myocarditis within four days after receiving an mRNA-based COVID-19 vaccine, predominantly in younger males after the second vaccine dose [5]. Successively, additional reports followed, suggesting a possible association between acute myocarditis and mRNA-based COVID-19 vaccination in healthy young adults and pediatric patients [6]. Given these premises, we provide here further details about these observations aiming at specifying their clinical profiles and pre/post-treatment cardiovascular magnetic resonance (CMR) findings.

## 2. Materials and Methods

This is a single-center, retrospective case series in which we collected six consecutive young patients (4 males and 2 females, 16 years old (Q1, Q3: 15, 18)) that experienced cardiac involvement after COVID-19 vaccination. The participants were enrolled between August and September 2021.

All patients were hospitalized due to fever and troponins elevation following the second dose of an mRNA-based COVID-19 vaccine. Cardiac magnetic resonance (CMR) (Excite, GE Medical System, Milwaukee, WI, USA) imaging was contextual with their hospitalization. We also provide their clinical profiles along with CMR findings. The follow-up ended at the time of the second CMR.

In agreement with the Declaration of Helsinki, all patients enrolled gave written informed consent during their evaluation, stating that data and images may be subsequently used for research purposes. For participants under 18 years old, a parent and/or the legal tutor gave informed consent. Since the study’s observational nature, ethical committee approval was not required.

### 2.1. Therapeutic Intervention

All patients were treated uniformly following the current European Society of Cardiology (ESC) Guidelines for the Diagnosis and Management of Pericardial Diseases [7].

Ibuprofen (Famar S.A.—Anthousa Attiki, Grece) was administered at 600 mg every 8 h for 2 weeks. Next, the ibuprofen was tapered by 200 mg every 1 week and then stopped. Colchicine (Special Product’s Line S.p.A.—Anagni, Italy) 0.5 mg once (<70 kg) or 0.5 mg b.i.d. (≥70 kg) was administered for the whole ibuprofen treatment.

After these 3 months, all patients underwent a follow-up CMR examination.

### 2.2. Cardiovascular Magnetic Resonance Imaging

A 1.5 T CMR scanner, equipped with a high gradient field strength (53 mT/m) and a four-channel cardiac phased-array coil, was utilized for all the exams. At the baseline and follow-up, multislice multiplanar unenhanced double inversion recovery (IR) fast spin-echo (FSE) T1- weighted, triple IR (Triple-IR) FSE T2-weighted static images and fully balanced steady-state sequence (FIESTA-GE Medical System) were acquired for morphological and functional evaluations. Then, all the patients received an intravenous bolus injection (0.05 mmol/kg; infusion rate 5 mL/s) of gadolinium chelate (Gd-DOTA, Dotarem, Guerbet, Paris, France). Late gadolinium-enhanced images (LGE) were acquired 12-to-15 min after contrast injection, with a series of 2D Fast IR with T1 images ranging between 220 and 250 to null normal myocardium signal.

Myocarditis was defined by the presence of an LGE typical pattern (subepicardial or patchy associated with hyperenhancement in T2W images). Myopericarditis was defined by the presence of the CMR findings mentioned above, associated with pericardial effusion [7].

### 2.3. Statistical Analysis

Categorical variables are presented as frequency rates and percentages. Continuous variables are presented as mean and standard deviation (SD) or median and 25th and 75th percentiles (Q1, Q3), where appropriate. Percentages were calculated by dividing the total by the number of subjects, and then multiplied by 100.

## 3. Results

Considering the current number of vaccinations administered in the Marche region, Italy (*n* = 231,989 www.regione.marche.it/Entra-in-Regione/Vaccini-Covid/Dati-aggiornati accessed on 30 December 2021) [3] and that our hospital is the hub of this region for cardiovascular diseases, we documented and reported a minimal amount of myocarditis/myopericarditis mRNA-COVID-19-vaccination related. This quantity cannot be utilized for a rate/prevalence analysis since it refers only to the patients who gave informed consent and were hospitalized but can be helpful to contextualize the disproportion of events if compared to the doses administrated.

Excluding the atrial tachycardia, no significant ECG abnormalities were found at the presentation. No significant echocardiographic abnormalities were documented. Considering echocardiography, only pericardial effusions were recorded in female participants.

All the CMRs were performed 72–96 h after receiving the second dose of an mRNA-based COVID-19 vaccine. The latter was administered 64 ± 29 h before the symptom’s appearance. Females were both hospitalized 96 h after vaccination. Four out of six patients were immunized with Pfizer-BioNTech and two with Moderna vaccine. The median in-hospital stay was 7 ± 2 days. All the post-treatment CMRs were performed after a median time of 3 ± 0.5 months.

The participants’ general, anthropometric, laboratories, and demographic characteristics are summarized in Table 1.

### 3.1. Clinical Characteristics and Laboratory Findings

The median high-sensitive Troponin-I (Hs-TnI) at the Hospital admission was 2373 (Q1, Q3: 576, 8123) ng/mL, while the c-reactive protein (CRP) was 4 ± 1.8. Only one patient had a history of AVNRT and on-treatment with Atenolol. No BNP increment was acutely found.

### 3.2. Cardiac Magnetic Resonance Findings

**Figure 1:**
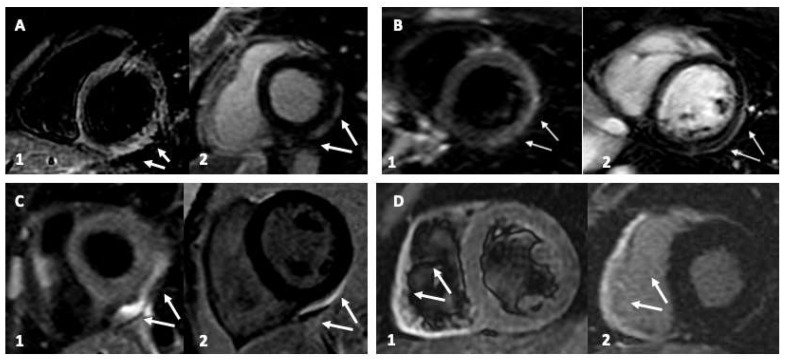
Cardiac magnetic resonance (CMR) imaging of young patients with vaccine-related myocardial involvement. Panel (**A**,**B**): Inferolateral myocardial involvement with edema and late gadolinium enhancement (LGE), with a subepicardial and pericardial distribution typical of myocarditis. Panel (**C**): Only pericardial involvement. Panel (**D**): isolated right ventricle involvement. Sub-Panel 1, triple IR (Triple-IR) FSE T2-weighted images for myocardial edema detection. Sub-Panel 2, LGE images.

All the pertinent CMR findings are summarized and presented in Table 2 according to participants. Myocarditis was prevalent in males (65% (*n* = 4/6)). It was characterized by myocardial edema (T2w hyperenhancement) and LGE in the lateral wall of the left ventricle (LV). Only one patient showed an isolated right ventricular (RV) involvement, while the females presented prevalently with myopericarditis (30% (*n* = 2/6) (Figure 1). All patients in our series demonstrated preserved LV ejection fraction and remained clinically stable during a relatively short in-hospital stay.

### 3.3. Follow-Up

**Figure 2:**
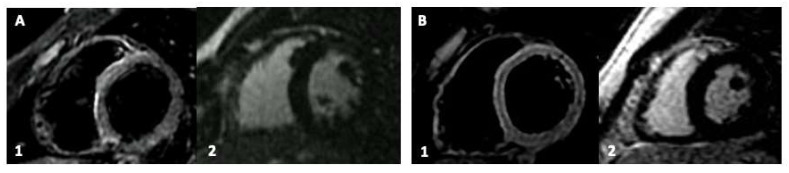
Cardiac magnetic resonance (CMR) imaging at the follow-up after medical treatment of young patients with vaccine-related myocardial involvement. Panel (**A**,**B**): Participants with previous inferolateral myocardial involvement. Sub-Panel 1, Short-Tau Inversion Recovery (STIR) images for myocardial edema detection. Sub-Panel 2, late gadolinium enhancement (LGE) images.

After a median follow-up time of 3.0 ± 0.5 months, all the patients are alive and asymptomatic. The CMRs performed after the ibuprofen and colchicine treatment did not present persistent cardiac involvement (Figure 2). No CRP or Hs-TnI elevation was found. The patient that experienced atrial tachycardia was acutely and effectively treated with metoprolol and did not show further arrhythmias at the 24 h ECG Holter-monitoring, suggesting a self-resolution.

## 4. Discussion

In our series, myocarditis was more prevalent in males, characterized by myocardial edema and LGE in the lateral wall of the left ventricle (LV). One patient presented lone RV involvement, while the females had myopericarditis. All patients had preserved LV ejection fraction and remained clinically stable during a relatively short inpatient hospital stay. One case presented with atrial tachycardia. After treatment, according to the current ESC guidelines, no cardiac involvement was detected at CMR imaging.

Previously published case series and reports already described possible cardiac involvement after COVID-19 vaccination [2]. Our report adds to previous literature, providing a detailed insight into clinical and CMR characteristics of COVID-19-related cardiac injuries. In addition, we also described a RV isolated myocarditis involvement. The latter correlates with more events and arrhythmias in the general population with myocarditis. Since it is difficult to be detected by standard echocardiography, if not promptly recognized and reported by imagers, an RV involvement may be overlooked and not treated by physicians adequately [8].

The diagnosis of myocarditis is challenging and typically based on clinical, electrocardiographic, and echocardiographic findings, elevated troponin levels, and a typical pattern on CMR [9,10]. A definite diagnosis cannot be conclusive without endomyocardial biopsy (EB) [11], even if the CMR diagnosis is increasingly accepted as highly convincing, especially in the pediatric population [12].

Myocarditis resulting from an enterovirus or human herpesvirus (HHV4 and HHV6) infection is commonly more severe in young and male patients. This variety of myocarditis can be associated with an immune–genetic background that increases the generation of autoantibodies and hormone-related factors contribute to the sex-specific differences observed in non-COVID-19 viral myocarditis [5,6,7,8,9,10,11,12,13]. Accordingly, in COVID-19 mRNA-vaccination-related myocarditis, the immune system might detect the mRNA in the vaccine as an antigen, resulting in the activation of pro-inflammatory cascades and immunological pathways in the heart [5]. Nevertheless, the gender difference that we observed in our series could be partially also explained due to the more extended period before the hospitalization of females.

Although myopericarditis after vaccination has been occasionally reported, even in the pre-COVID-19 era [14], our series highlights the benign nature of the mRNA COVID-19 vaccines-induced myocarditis and myopericarditis. In addition, we demonstrated that such complications result in a relatively short period of hospitalization, at a modest rate of trivial complications, that can be effectively treated with a standard medication treatment with ibuprofen and colchicine, without any CMR-detectable consequence. In addition, the benefit-risk for COVID-19 vaccination has been already proven, showing a favorable balance for vaccination in all age and sex groups [4].

Otherwise, we believe that additional studies and multicentric registry are needed and encouraged to clarify the incidence, risk factors, genetic predisposition, prognosis, potential mechanisms, reasons for sex differences, clinical course, treatment strategies, and the long-term impact of myocarditis after COVID-19 vaccination.

## 5. Conclusions

According to other recently published case series, our series suggests a possible association between sporadic acute myocarditis/myopericarditis cases and mRNA COVID-19 vaccination in healthy young adults and pediatric patients. Not only males are involved, while some arrhythmic manifestations are possible. Conversely, we here highlight the benign nature of such complications and the absence of CMR findings after a three-month medical treatment with colchicine and ibuprofen.

## Figures and Tables

**Table 1:** General characteristics of patients hospitalized due to vaccine-related myocardial involvement at the CMR imaging.

Patient	Age ^1^	Sex ^2^	Vaccination	History	Medication	Temp ^3^	HR ^4^	BP ^5^	CRP ^5^	Adm Hs-TnI ^6^	Nadir Hs-TnI ^6^	BNP ^7^
1	17	F	Pfizer/BioNTech	AVNRT	Atenolol	37.1	98	110/70	2.2	439	6609	73
2	18	M	Pfizer/BioNTech	RBBB	//	38.2	70	120/80	4.3	8123	9897	57
3	25	M	Moderna	//	//	38	57	120/70	7.3	40568	12500	5
4	16	F	Moderna	//	//	37.6	77	125/65	3.5	576	5703	76
5	15	M	Pfizer/BioNTech	//	//	38.3	78	115/67	4.5	4070	6078	58
6	14	M	Pfizer/BioNTech	//	Acetaminophen	37.7	69	123/70	2.7	676	5047	45

Legend: Temperature (Temp); heart rate (HR); blood pressure (BP); c-reactive protein (CRP); admission (Adm); high-sensitive Troponin-I (Hs-TnI); Brain Natriuretic Peptide (BNP); atrioventricular nodal reentrant tachycardia (AVNRT); right bundle branch block (RBBB); upper limit (upl). Footnotes: ^1^ (years); ^2^ (M/F); ^3^ (C°); ^3^ (bpm); ^4^ (mmHg); ^5^ (mg/dL), upl 0.6; ^6^ (ng/L), range: Females 2–51, males 2–75; ^7^ (pg/mL), upl 100.

**Table 2:** General characteristics of patients hospitalized due to vaccine-related myocardial involvement at the CMR imaging.

Patient (n°)	LV EDV ^1^	CMR EF ^2^	LGE ^3^	LGE Distribution	Triple-IR ^3^	FU-LGE
1	67	55	y	Pericardium	y	n
2	75	65	y	Inferolateral	y	n
3	77	57	y	RV	y	n
4	62	62	y	Pericardium	y	n
5	67	60	y	Inferolateral	y	n
6	65	67	y	Inferolateral	y	n

Legend: Number (n); end-diastolic volume (EDV); late gadolinium enhancement (LGE); triple IR (Triple-IR) FSE T2-weighted; follow-up (FU); yes (y); no (n). Footnotes: ^1^ (mL); ^2^ (%); ^3^ (y/n).

## Data Availability

All data relevant to the study are included in the article.
